# Two year remission rates and functional outcomes in an inception cohort of Canadian children with juvenile idiopathic arthritis

**DOI:** 10.1186/1546-0096-10-S1-A60

**Published:** 2012-07-13

**Authors:** Shirley ML Tse, Lori B Tucker, Gaelle Chédeville, Janet E Ellsworth, Jaime Guzman, Kim Morishita, Rosie Scuccimarri, Natalie J Shiff, Ciaran M Duffy, Rae SM Yeung, Kiem G Oen

**Affiliations:** 1 BC Children's Hospital, Vancouver, BC, Canada; 2 Montreal Children's Hospital, Montreal, QC, Canada; 3 Royal University Hospital, Saskatoon, SK, Canada; 4 Stollery Children's Hospital, Edmonton, AB, Canada; 5 The Hospital for Sick Children, Toronto, ON, Canada; 6 UBC and BC Children's Hospital, Vancouver, BC, Canada; 7 University of Manitoba, Winnipeg, MB, Canada

## Purpose

To examine the remission rates and functional outcomes in an inception cohort of patients with juvenile idiopathic arthritis (JIA).

## Methods

The Research in Arthritis in Canadian Children, emphasizing Outcomes (ReACCh-Out) cohort is a 16-centre inception cohort of patients with JIA enrolled within 1 year of diagnosis. All patients who completed 5 six-monthly study visits from baseline to 24 months were included in this analysis. Disease activity and functional measures were collected at each visit. Criteria for disease remission status in this study were defined by Wallace et al (2006) modified by omission of ESR or CRP due to incomplete data. Inactive disease (ID) was defined as no active joints, physician global assessment 0, and no active extra-articular disease; clinical remission on medications (CRM) is ID for ≥ 6 months on medications; and clinical remission (CR) is ID for ≥ 12 months off medications. Health status was measured using the Juvenile Arthritis Quality of Life Questionnaire (JAQQ), and functional status using the Child Health Assessment Questionnaire (CHAQ).

## Results

Data for 347 of 1574 patients enrolled in the ReACCh-Out cohort was available for analysis. Median age of disease onset was 7.5 yr (IQR 3,11), with 35% oligoarticular, 22% polyarticular RF negative, 13% enthesitis related arthritis, 8% systemic, 8% undifferentiated, and 7% for each polyarticular RF positive and psoriatic subtypes. The percentage of patients with ID was 33% at 6 months, 39% at 12 months and 49% at 24 months; CRM increased from 4% at 6 months to 18% at 12 months and 26% at 24 months, while CR at 24 months was 7%. Differences in remission rates were evident amongst the JIA subtypes by 12 months, with the highest remission rates in oligoarthritis and lowest in the RF positive polyarthritis (CRM of 29% and 4% respectively). Even when ID was not reached, a decrease in active joint count was noted in all JIA subtypes. CHAQ and JAQQ scores improved in all patients as early as 6 months. Differences were also evident amongst the JIA subtypes with the best 24 month median scores in oligoarthritis patients (CHAQ=0, JAQQ=1.35) compared with the worse scores in RF+ polyarthritis patients (CHAQ=0.625, JAQQ=2.35).

## Conclusion

While active joint counts and functional outcomes improved within the first 24 months, disease remission rates differed amongst JIA subtypes, and CRs are rare. The remission rates derived from our inception cohort of JIA patients will assist in family counseling, pinpoint subgroups requiring aggressive therapy and inform the design of clinical trials.

## Disclosure

Shirley M.L. Tse: None; Lori B. Tucker: None; Gaelle Chédeville: None; Janet E. Ellsworth: None; Jaime Guzman: None; Kim Morishita: None; Rosie Scuccimarri: None; Natalie J. Shiff: None; Ciaran M. Duffy: None; Rae S.M. Yeung: None; Kiem G. Oen: None.

